# Human Embryo Models and Drug Discovery

**DOI:** 10.3390/ijms22020637

**Published:** 2021-01-11

**Authors:** Margit Rosner, Manuel Reithofer, Dieter Fink, Markus Hengstschläger

**Affiliations:** Center for Pathobiochemistry and Genetics, Institute of Medical Genetics, Medical University of Vienna, 1090 Vienna, Austria; margit.rosner@meduniwien.ac.at (M.R.); manuel.reithofer@meduniwien.ac.at (M.R.); dieter.fink@meduniwien.ac.at (D.F.)

**Keywords:** human pluripotent stem cells, human embryonic stem cells, organoid, embryoid, gastrulation, disease modelling, drug discovery

## Abstract

For obvious reasons, such as, e.g., ethical concerns or sample accessibility, model systems are of highest importance to study the underlying molecular mechanisms of human maladies with the aim to develop innovative and effective therapeutic strategies. Since many years, animal models and highly proliferative transformed cell lines are successfully used for disease modelling, drug discovery, target validation, and preclinical testing. Still, species-specific differences regarding genetics and physiology and the limited suitability of immortalized cell lines to draw conclusions on normal human cells or specific cell types, are undeniable shortcomings. The progress in human pluripotent stem cell research now allows the growth of a virtually limitless supply of normal and DNA-edited human cells, which can be differentiated into various specific cell types. However, cells in the human body never fulfill their functions in mono-lineage isolation and diseases always develop in complex multicellular ecosystems. The recent advances in stem cell-based 3D organoid technologies allow a more accurate in vitro recapitulation of human pathologies. Embryoids are a specific type of such multicellular structures that do not only mimic a single organ or tissue, but the entire human conceptus or at least relevant components of it. Here we briefly describe the currently existing in vitro human embryo models and discuss their putative future relevance for disease modelling and drug discovery.

## 1. Animal Models and Immortalized 2D Human Cell Culture Models

It has always been a fundamental endeavor in biomedical research to improve the quality and efficiency of the experimental approaches to investigate human pathologies. Whether with respect to viral or bacterial infectious diseases, monogenetic pathologies or the wide variety of multifactorial diseases, the ultimate research aims are the elucidation of the underlying molecular mechanisms, the identification of prognostic and diagnostic factors and the discovery of new therapeutic targets and drugs. The inaccessibility of the in vivo human condition, the scarcity of human tissue material for research, ethical concerns, and legal constraints made it essential to establish appropriate models to mimic and study human maladies. During the past decades animal model systems and immortalized two-dimensionally (2D)-cultured cell lines, often derived from tumor specimens, were the only options of choice. Still, it is important to note that such models have successfully been used to gain basic pathophysiological insights, to identify potential drug targets and to design and test new candidate drugs for many diseases. However, for different reasons, the historical reliance on these two major disease modelling approaches also often did not allow the development of a comprehensive understanding of the molecular basis and mechanisms underlying specific human pathologies and the establishment of new therapeutic concepts [1].

The evolutionary conservation of many fundamental biological mechanisms made it possible to obtain an elaborated understanding of the molecular development of pathologies by studying animal model organisms. Mainly because of their robustness, their potential to grow fast, their high reproduction rate and the possibility to propagate them at low costs made yeast (*Saccharomyces cerevisiae*), the worm (*Caenorhabditis elegans*), the fruit fly (*Drosophila melanogaster*), zebrafish (*Danio rerio*), and the mouse (*Mus musculus*) to the most commonly used experimental models. Compared with each other and with cancer cell lines or patient-derived xenografts, commonly used in oncological research, all these models have both, unique advantages and specific limitations. Accordingly, depending on the scientific question of interest, the appropriate model system must also be chosen considering different aspects, such as, for example, ease of maintenance, duration of experiments, suitability for genetic manipulations or genome-wide screening approaches, or their relative costs [2]. Although these model systems share the features of being amenable to experimental manipulation and convenient to investigate, they also exhibit particular shortcomings, when the obtained results should be translated to the human condition.

Due to species-specific differences in physiology, metabolism, and genetics, animal models are by their nature imperfect tools to study human physiology and pathophysiology [3,4]. Still, especially genetic modifications in mice provided relevant insights for the understanding of the molecular development and the progression of different human genetic diseases. On the other hand, results obtained from mouse studies cannot necessarily be extrapolated to the human condition. This is of special relevance for tissues and organs, known to develop via different processes and mechanisms in mice and men. For example, different molecular and cellular events and mechanisms trigger the development of the human brain, which is far more complex than its mouse counterpart [2,5,6,7]. The importance to investigate human brain cells to draw conclusions on the situation in men was recently supported by a single-cell RNA sequencing study describing extensive differences between homologous human and mouse cortex cell types, including marked alterations in proportions, laminar distributions, gene expression, and morphology [8]. Another prominent example for limitations concerning cross-species comparisons is the mammalian embryogenesis. The substantial morphological and biological differences between human and mouse embryonic development have already intensely been studied [9,10,11]. Still, rodent animal models have been very useful tools for the discovery of new targets and testing innovative therapeutic drugs. Nevertheless, although studies with such animal models led to promising results in certain preclinical trials, the same treatments do not always translate to human clinical trials. According to the U.S. Food and Drug Administration (FDA) 80 to 90% of drug candidates entering phase I clinical trials fail due to a lack of sufficient efficacy and/or because of toxicity. A central goal for biomedical researchers and patients alike is, thus, the efficient and reliable identification of drugs with high efficacy and low toxicity. Since the indispensable clinical trials are time-consuming and costly, it is of highest relevance to continuously improve their success rate [1,12,13]. The translation of preclinical findings in mice to clinical trials in humans is impaired by species-specific differences concerning a variety of factors, such as, for example, the permeability of the drug candidate, the physiology of the intestinal tract, or polymorphisms in drug-metabolizing enzymes. The latter is of special interest, also because it is known that drug metabolism in mice is regulated by 34 cytochrome P450 gene families, whereas in humans only eight are major participants [14,15]. Accordingly, it is not surprising that these currently used preclinical models are not always sufficiently able to detect drug associated toxicities. A summary of the results of a multinational pharmaceutical company survey and the outcome of an International Life Sciences Institute Workshop in April 1999 revealed that 57% of 221 human toxicity events caused by 150 compounds were not predicted by preclinical rodent studies [16]. Due to such prediction problems but also because of ethical concerns regarding animal work, it is an already long lasting and still ongoing discussion in various countries to which extent academic research institutions and pharmaceutical companies, including those developing cosmetics, but also those working on human drug development, should avoid the use of animals especially in preclinical drug-testing approaches. Ethical concerns were particularly discussed in the context of research using animal models, such as non-human primates. For example, infectious diseases have been studied in non-human primates in the past. However, besides the ethical aspects it is also important to keep in mind that conservation of gene functions, molecular processes, and physiological regulations with the human context is not guaranteed [17,18,19,20].

Traditional human cell lines represent one cell type, either floating as single cells in suspension cultures or growing attached to the culture dish. This can, by far, not recapitulate the in vivo organ architecture and physiology with its cell–cell interactions and does never allow a comprehensive understanding of human maladies, which always develop in complex multicellular ecosystems. Furthermore, these cell lines are usually immortalized and/or transformed associated with the induction of genomic instability, either because there are derived from human tumors or their indefinite in vitro propagation was enabled by transfection with viral oncogenes [3,21]. The time-consuming process of genetic and phenotypic adaption of such cells to culture conditions is rewarded by the establishment of a rapidly growing and cheap cellular model that is easy to handle and amenable to a variety of technological approaches. However, these models exhibit clear limitations regarding their potential to mimic real in vivo human conditions including for example the interaction of cells of different origins [22,23].

In the last years, the interest in humanized animals as models for cancer research constantly increased. Mice can provide a platform for the evaluation of the tumor microenvironment, when they are engrafted with components of the human immune system and implanted with tumorigenic cell lines or in the form of xenografts derived from patients [24]. In patient-derived xenograft models (PDX), known already since their first successful establishment in 1953 [25], primary human tumor tissue is transplanted into immune-deficient mice. Since such approaches allow the preservation of the tumor structure and the relative proportion of tumor and stromal cells, tumor complexity and heterogeneity are clearly better retained than in cell lines. Likewise, gene therapies for monogenetic diseases using gene editing approaches, but also for example chimeric antigen receptor (CAR) T cell therapies for cancer, will also benefit from humanized animal models regarding the assessment of their efficacy and safety prior to human clinical trials [26]. However, the establishment of such models is still inefficient, their applicability for investigations of human pathologies is limited and high-throughput analyses are laborious and expensive [24,26,27,28].

## 2. Human Pluripotent Stem Cells

With the advent of the establishment of isolation protocols and culture conditions for human pluripotent stem cells (hPSCs), including human embryonic stem cells (hESCs) [29] and human induced pluripotent stem cells (hiPSCs) [30], the era of the so-called hPSC-based drug discovery (hPDD) was initiated [31]. Pluripotent stem cells are per definition cells that express markers of pluripotency, can proliferate indefinitely while maintaining cellular identity (self-renewal) and are able to differentiate into cells of all three embryonic germ layers [32]. Interestingly, hESCs require different culture conditions compared to mouse embryonic stem cells (mESCs). Gene expression studies revealed that mESCs and hESCs display characteristics of the pre-implantative inner cell mass and the post-implantation epiblast, respectively. Accordingly, pluripotency is considered to exist in two states: naïve, represented by mESCs; and primed, typical for conventional hESCs. mESCs and hESCs share the potential to contribute towards all embryonic derivates but are limited in their potential to develop into extraembryonic tissue [33]. Their tri-lineage differentiation potential together with their non-transformed status are obvious advantages of hPSCs over conventional immortalized human cell culture models. These inherent characteristics of hPSCs including the fact that stem cells are perfectly amenable to genetic modifications [34,35] make them a powerful tool for both, studies on the molecular basis of human diseases and hPDD [4,31,36,37,38]. On the other hand, as for most other cell types, traditional 2D cultivation of hPSCs and their differentiated derivatives exhibits inevitable technological limitations and conceptual biases. Although since the year 2000 widely used, no significant drug discoveries were made by hPDD in its beginnings. It was a long way paved with different technological improvements that finally enabled a substantial reduction of experimental variability with a concomitant increase in reproducibility.

In between, in a variety of studies, especially hiPSC-derived cells have been used for testing the efficacy and toxicity of different compounds [34,37]: hiPSC-derived cortical neurons were used to test the impact of 3838 compounds in Lesch-Nyhan disease [39] and 17,000 small molecules were tested for their anti-fibrotic effects in mesenchymal-like cells [40]. hiPSC-derived neurons, neural progenitor cells, and motor neurons have been generated to study the effects of the R33 molecule for Alzheimer’s disease, the effects of 135 compounds for Schizophrenia, and 1416 drugs for Amyotrophic lateral sclerosis, respectively [41,42,43]. With the aim to establish new therapeutic concepts for familial hypercholesterolemia 2320 drugs were tested on hiPSC-derived hepatocytes [44], and just to mention another example, cardiomyocytes were developed from iPSCs to test whether the compound LUF7346 can rescue some phenotypic features of Long-QT-syndrome [45].

Still, until now the successful application of hPSCs in drug investigations is precluded by different limitations. The disease models established from hPSCs do not allow a complete recapitulation of all characteristics of a human pathology, and next to the question of the choice of the appropriate differentiated cell type for the drug screening design, the heterogeneity and genomic instability arising from extended cultivation and differentiation procedures still limit the application of hPSCs in drug discovery [31]. With regard to, e.g., gene expression levels or epigenetic patterns, the variation across different hPSC lines is known to be high. In order to minimize heterogeneity and experimental variability in 2D drug screening approaches, the application of single-stem cell-based non-colony type monolayer cultivation is required. These cultivation procedures are based on single cell dissociation of hPSC colonies and the prevention of dissociation-induced apoptosis in the presence of inhibitors of the Janus kinase 1 or the Rho-associated protein kinase. The extended inhibitor treatment, known to be necessary for 2D non-colony type stem cell cultures, might affect the drug screening process [46,47], and most importantly, cells in the human body never fulfill their functions in mono-lineage isolation and human diseases always develop in complex multicellular ecosystems [22,23,31,37]. In this context, hPSCs are known to interact with their environment via different modes of paracrine signaling [48,49]. Accordingly, for the optimization of drug discovery it was of highest relevance to establish multi-lineage three-dimensional (3D) culture conditions, which allow a more accurate in vitro recapitulation of human physiology and pathophysiology.

## 3. Human Organoids

In addition to xenograft approaches, many different cell culture models, including, for example, spherical cellular aggregates (known as spheroids), have successfully been established which meet the criteria of being 3D. Some of these spheroid models, such as, for example, mammospheres or neurospheres, can recapitulate specific in vivo conditions and allow to investigate some aspects of cell interactions, what makes them suitable for preclinical drug testing. Tumor spheroids can account for basic features of solid tumors and, when co-cultured with other cells, can mirror human tumor–stroma interactions. Still, since these 3D cellular structures consist of only very specific cell types, their applicability for pharmacological research is limited [50,51,52].

Under certain suspension culture conditions hPSCs can develop into 3D aggregates, called embryoid bodies (EBs), recapitulating some aspects of early embryogenesis. During this specific differentiation approach endoderm forms on the surface of the EBs, what is followed by the formation of a central cavity and the development of a columnar epithelium with a basal lamina. During EB formation rather homogeneous epithelial-like hPSC colonies undergo epithelial-mesenchymal transition (EMT) and develop into 3D structures containing interacting cells indicative of the ectoderm, mesoderm and endoderm. Although in principal, EBs can be used for target identification and drug testing, one must be aware that they lack controlled organization of differentiation or tissue development and do neither recapitulate a realistic in vivo condition nor a specific stage or morphological structure of an embryo [20,53,54,55,56].

All the knowledge and technical experience obtained with 2D and 3D cell cultivation procedures, cell differentiation approaches, bio-printing or cultivation in microfluidic devices (organ-on-a-chip) finally resulted in the kick-start of the field of organ-like miniatures to study normal and abnormal biological processes [57,58,59]. The so-called organoids (meaning “resembling an organ”) are now defined as self-organizing 3D structures grown from pluripotent or adult stem cells, which mimic the in vivo architecture and multi-lineage differentiation of the original tissue in mammals [60]. Although a variety of different studies already had provided essential basic requirements, this field was not really opened before the groundbreaking demonstration of organoid development from intestinal adult stem cells [61] and the pluripotent stem cell-based in vitro development of cortical tissues and the optic cup [62,63]. Since organoids are self-organizing, they require minimal external manipulation and are in principle technically reproducible [59]. Organoids can be derived or generated from healthy human cells, maintain stable genotypes and develop to phenotypes, similar and sometimes histologically indistinguishable from actual human organs [2,64,65]. Due to all these characteristics, organoids provide optimal tools for basic biological research as well as for target identification and drug discovery. In combination with modern gene editing technologies [66,67,68,69] organoids will permanently contribute to the molecular understanding of human genetic diseases in future. Until today, hPSC-derived intestine, lung, stomach, thyroid, liver, kidney, blood vessels, and brain organoids and human adult stem cell-derived intestine, lung, pancreas, endometrium, stomach, prostate, and liver organoids have already successfully been established [2,3,4,22,23,57,58,59,60,64,65], and the field is growing rapidly. It is important to note that until now the observed heterogeneity of the generated organoids is still a relevant limitation. In addition, compared to the corresponding organs they are of different scale and they are far less complex. In contrast to organoids, in vivo organs interact with the local environment within the body in a network of nerves and blood vessels and are populated with microorganisms.

However, there are already many examples for the usage of organoids in modelling of certain diseases and for drug discovery [3,23,37]. Using material derived from patients, organoids modelling for example cystic fibrosis [70,71], Rett syndrome [72], α1-antitrypsin deficiency [73], polycystic kidney disease [74,75], or Hermansky-Pudlak syndrome [76] have already been established. Furthermore, a variety of different disease modelling organoids have been generated using hiPSCs derived from patients carrying specific diseases-associated mutations: Among many others, retinal organoids modelling retinitis pigmentosa [77], colon organoids modelling colorectal cancer [78], and hiPSC-derived intestinal organoids from cystic fibrosis patients [79] were established. Patient-specific hiPSC-derived brain organoids can be used to model for example lissencephaly [80] or Down syndrome [81]. Organoids have also successfully been established to investigate host-microbe interactions in infectious diseases, such as replication of norovirus in human intestinal stem cell-derived enteroid culture [82]. The pathological consequences of Zika virus infections have been mimicked and studied in brain organoids [83,84]. Forebrain organoids have also already been useful in drug discovery. They enabled the identification of small-molecule inhibitors of Zika virus infection and induced neural cell death [85]. hPSC-derived intestinal organoids have been shown to enable investigations on the consequences of common viral infections and infection with pathogenic microorganisms [86,87,88]. Lung organoids have for example successfully been used to study infections with viruses, including emerging influenza strains [89,90], and finally, cystic fibrosis is an example where organoid-based drug testing is already routinely used in clinical practice, so that Dutch health insurers pay for this individualized diagnosis. Organoids generated from patient-derived tissue allow to estimate whether a specific drug will be effective in this patient [91].

## 4. Human Embryoids

Independent of whether they are derived from adult stem cells or from hPSCs, organoids are typically the in vitro recapitulations of one specific lineage, tissue, or organ. The recent developments regarding the establishment of hPSC-based embryo models, called embryoids (or embryo-like structures, synthetic embryos or synthetic entities with embryo-like features) allow the investigation of the self-organizing mechanisms in multi-lineage settings. All reported human embryo models are multicellular structures mimicking the development and homeostasis of different relevant components of the human conceptus. The hPSCs used to generate embryoids are well established and their cultures are robust. Since these embryo models develop and mature within only a few days, they also exhibit a more reproducible cellular organization than organoids [10,11,92,93,94,95].

Although these characteristics would qualify them as tools for drug discovery, embryoids are currently still primarily used to investigate the very first events in human development. So far, most important insights into the early steps of mammalian embryogenesis originate from mouse studies. However, as already mentioned above, conclusions drawn from investigations on mouse embryos cannot necessarily be assigned to humans. The pre-implantation development from a zygote to a blastocyst, with a trophectoderm enclosing a cavity and an inner cell mass, is conserved between mice and men. The same is true for the further segregation of the inner cell mass into the pluripotent epiblast and the primitive endoderm. However, both the timing of the blastocyst implantation and the post-implantation morphogenesis and lineage development, show significant interspecies differences [9,10,11,96].

The studies performed on surgery-derived histological specimens decades ago provided only limited insights into early human development and are nowadays considered unethical in most countries. Undoubtedly, the collection of snapshots of human embryos held at the Carnegie Institution of Washington is still of high scientific relevance for the understanding of the morphogenic processes governing early development. However, by nature, it does not allow conclusions regarding the molecular mechanisms underlying human embryogenesis [33,92,97].

With the advent of in vitro fertilization (IVF) additional insights into human embryogenesis were enabled by prolonged cultivation of embryos left over from IVF [92,95,98,99,100]. However, following the Warnock 14-d rule implemented in 1984, the legislation of many countries allows researchers to maintain vital human embryos only until day 14, prohibiting investigations of post-implantation processes such as primitive streak development and gastrulation [101,102]. Still, culture platforms for the growth of surplus IVF human embryos up to day 14, could pave the way to the modelling of infections and drug discovery. Recently, such platforms were used to analyze the gene expression of *ACE2*, encoding the SARS-CoV-2 receptor, and *TMPRSS2*, encoding a protease that cleaves the ACE2 receptor and the viral spike protein to facilitate infection. The demonstrated expression of these genes in the trophoblast of the blastocyst and syncytiotrophoblast and hypoblast of the implantation stages indicates that embryos/embryo-like structures could allow further investigations of the potential risks of putative vertical virus transmission to implantation and fetal health [103]. It is important to note that ethical and technical barriers hinder the application of genetic modification approaches using human embryos, and the fact that many embryos donated from IVF patients harbor aneuploidies or gene mutations is an additional limiting aspect for investigations of the molecular processes regulating early human development. Studying embryogenesis of non-human primates is also not considered a reasonable alternative, because of ethical concerns, legal constraints, and the fact that conservation of molecular processes is not guaranteed [20].

Accordingly, the wide research field recently initiated by the successful implementation of protocols to generate embryoids is expected to have enormous impact for a more comprehensive understanding of the early steps of human embryogenesis. The currently existing in vitro human embryo models include 2D gastrulation micropatterned colonies [104,105], asymmetric early post-implantation epiblasts [106], post-implantation amniotic sac embryoids (PASE) [107,108,109,110], and the very recently described 3D gastruloids [111].

### 4.1. Gastrulation Micropatterned Colonies

**Figure 1:**
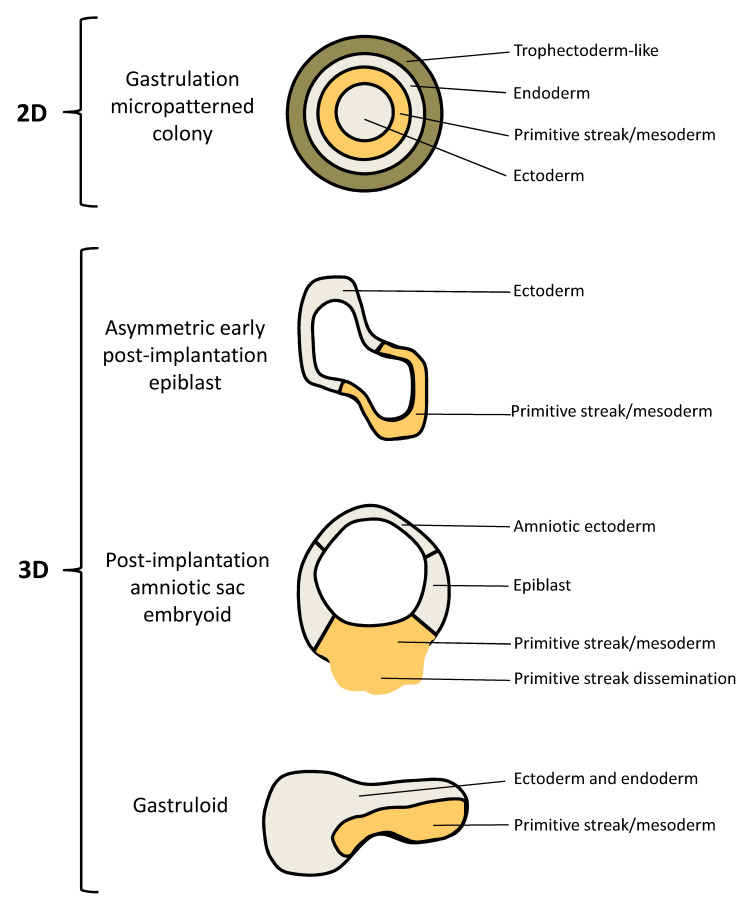
Schematic representation of the human embryo models. For detailed description see the text and Table 1.

hESCs (which normally grow without defined organization, when cultivated in conventional 2D environments) confined to circular micropatterns (on slides with arrays of disks where extracellular matrix (ECM) proteins bind and control cell adhesion) and treated with BMP4 have been demonstrated to produce an ordered array of germ layers equivalent to the human gastrulating embryo. The so induced self-organized patterning of hESCs triggers the development of embryoids, also referred to as human 2D gastruloids. These embryo-like structures consist of an outer trophectoderm-like, extra-embryonic-like ring, which is positive for CDX2 and negative for Brachyury (BRA), SOX17, and SOX2; a center composed of SOX2+/NANOG-/BRA-/SOX17- ectodermal cells; and SOX17+/NANOG+/SOX2− endodermal and BRA+/NANOG+/SOX17−/SOX2− mesodermal cell layers in between (Figure 1; Table 1). It was shown that the distance from the colony edge influenced cell fate, coinciding with the localization of BMP4 receptors. Cells in the primitive streak-like region can undergo EMT and migrate inwards toward the surface of the culture dish mimicking gastrulation movements [104,105]. The artificial 2D structure of this model is an obvious disadvantage when one wants to translate results obtained studying these gastruloids to the in vivo 3D condition. In the human embryo the three germ layers are positioned on top of each other, with a surrounding trophectoderm. The 2D micropatterned colonies do not resemble this in vivo morphology and, in addition, bilateral symmetry is not established in vitro. Furthermore, these synthetic structures lack key morphological features of the epiblast morphogenesis, such as, e.g., the formation of a central lumen to establish the pro-amniotic cavity. On the other hand, the ease of the experimental protocol and the reproducibility of the induced developmental features emphasize the relevance of this embryo model for qualitative and quantitative analyses of the mechanisms regulating germ layer patterning events. Consequently, this highly reproducible model is an optimal tool to investigate the molecular mechanisms controlling the self-organization potential of hPSCs. In future, its reproducibility will also call the attention of pharmacologist to deploying this embryoid model in target identification and drug discovery studies [11,20,93,112,113,114,115,116,117,118].

### 4.2. Asymmetric Early Post-Implantation Epiblasts

Since the architecture of 2D human gastruloids obviously deviates from the actual 3D in vivo embryo condition, a protocol for the establishment of a hESC-based 3D gastruloid was developed. In this model, hESCs dispersed in hydrogel supplemented with Matrigel and treated with a low dose of BMP4, form lumenal sacs (Figure 1; Table 1). These so called asymmetric early post-implantation epiblasts, whose size, cell polarity, and gene expression are similar to a day 10 human epiblast, allow the investigation of both polarization and lumen formation. This model exhibits polarization into two opposing regions displaying ectodermal (BRA−/SOX2+) and mesodermal (BRA+/SOX2−) gene expression patterns mimicking anterior-posterior symmetry breaking of the epiblast at the onset of gastrulation including EMT and the formation of primitive streak-like structures [106]. The obvious strengths of this spheroid model are the potential to recapitulate lumenogenesis and the property to mimic relevant aspects of spontaneous symmetry breaking. On the other hand, the developmental potential of these gastruloids has obvious limits since they, e.g., do not undergo amnion specification on one side of the lumen. Finally, asymmetric early post-implantation epiblasts lack the controllability and reproducibility of the 2D gastruloids. This is a relevant limitation with regard to screening approaches, either to identify the molecular regulators of the self-organization potential of hPSCs, or in the context of target identification and drug discovery investigations [20,93,94,95].

### 4.3. Post-Implantation Amniotic Sac Embryoids

The amniotic ectoderm is the first type of tissue differentiated from the epiblast during implantation. In 2017, a synthetic hESC-based 3D embryoid system was reported, which allows the in vitro differentiation and self-organization of the human amniotic ectoderm [107]. The same research group established the first model of peri- and post-implantation human amniotic sac development equivalent to the in vivo conditions until days 13–19 post fertilization. This PASE (post-implantation amniotic sac embryoid) model recapitulates multiple post-implantation embryogenic events centered around human amniotic sac development, which is drastically different from that in mice. In this model, hESCs placed onto a soft gel bed and covered with ECM-containing media, form an amniotic sac-like structure with amniotic ectoderm and the pluripotent epiblast enclosing the prospective amniotic cavity. Upon further development, the cells of the epiblast, which mimic the embryonic disc, start to differentiate into primitive streak followed by their dissemination into the 3D environment. The in vivo equivalents of the morphological and molecular key events modelled in the amniotic sac embryoid, including lumen formation, symmetry breaking and primitive streak development, are the formation of the epiblast cyst at days 6 to 7, asymmetric sac development at days 7 to 13 and the onset of gastrulation at days 13 to 19, respectively (Figure 1; Table 1) [108]. In this model, stable spontaneous symmetry breaking occurs in only 5–10% of the lumenal sacs. Recently, a microfluidic device has been established to increase the efficiency of PASE formation. This microfluidic approach allows the growth of clusters of hESCs in small indentations with separate channels supplying growth factor- and/or inhibitor-supplemented culture media to each side of the developing embryoid. This technology enables controllable and scalable studies on the development of the epiblast and amniotic ectoderm parts of the conceptus, including lumenogenesis of the epiblast, the development of a bipolar embryonic sac and the differentiation of primitive streak cells and primordial germ cells [109]. Furthermore, an amniotic ectoderm microtissue array platform for the quantitative phenotyping of lumenogenesis and amniogenesis of the epiblast has been established, and intriguingly, the application of this platform for drug screening was demonstrated. Six clinically relevant drugs were investigated for their differential toxicity on the amniotic ectoderm development: the common antibiotic Penicillin, which is regarded as safe for pregnancy; the teratogenic chemotherapeutic agent Doxorubicin (category D drug according to the FDA pregnancy categories); the two over-the-counter painkillers Ibuprofen and Acetaminophen, of which only the latter is recommended for use during pregnancy; the anti-inflammatory Dexamethasone and the anti-depressant Citalopram, for which no adequate and well-controlled studies in humans exist (category C drugs). This amniotic ectoderm microtissue array enabled successful detection of the differential teratogenicity of these drugs [110]. Accordingly, it is not surprising that the human amniotic sac model, including its variations, is already discussed as powerful tool for drug studies and toxicity screens [11,95]

### 4.4. Gastruloids

Very recently, the generation of hESC-based 3D gastruloids, exhibiting features of late Carnegie-stage 8 embryos (days 17 to 19) to early Carnegie-stage 9 embryos (days 19–21), has been reported. When hESCs are treated with Chiron, a glycogen synthase kinase 3 inhibitor functioning as an activator of the WNT pathway, before seeding into low-adherence plates, 3D multicellular aggregates are formed. Spatial transcriptomics demonstrated that these gastruloids exhibit patterned gene expression. These synthetic embryo models contain derivatives of all three embryonic germ layers organized in a spatiotemporal manner in the absence of extra-embryonic tissues. Distinct regions of the posterior elongated tip of these gastruloids express BRA (mesoderm), SOX17 (endoderm) and SOX2 (ectoderm), respectively. The anterior region is positive for markers of cardiac development. Overall, these embryoids are post-primitive streak structures with the transcriptional signature of somite formation (Figure 1; Table 1) [111]. Due to the legal boundaries for human embryo cultivation, ex vivo confirmation of results obtained with these gastruloids, which represent stages that lie beyond these boundaries, cannot be obtained. However, this is a matter of perspective, since this model provides an exclusive opportunity to study the dynamics of transcription, epigenetic patterns, signalling, and differentiation in the posterior human embryo beyond these boundaries. Without doubt, these synthetic embryos will enable new important insights into segmentation timing and transcriptional processes during somitogenesis. Furthermore, this model basically provides an opportunity for drug studies and toxicity screens in stages of human embryogenesis that lie beyond the boundaries defined by the legal regulations for ex vivo human embryo cultivation [20,33,111].

## 5. Conclusions

Although animal models and immortalized traditional 2D human cell cultures were, and still are, indispensable and powerful enablers of the progress in biomedical research, it was obviously imperative to establish a new generation of multi-lineage platforms for accurate evaluations of the interactions between multiple human cell types in the context of diseases as well as for drug discovery. Ideally, such new models should allow the recapitulation of the three-dimensional (3D) multicellular ecosystem of human organs, tissues, and pathologies and should be amenable to genetic modifications, genomic screens, personalized medicine approaches, cancer studies, modeling of infections, microbiome studies, target identifications, high-throughput screenings, and preclinical pharmacokinetic and pharmacodynamic drug testing.

In the recent past, it was mainly the convergence of two developments that had tremendous impact on the progression of the field of modeling human physiology and pathophysiology: the establishment of reliable procedures for the derivation and in vitro cultivation of hPSCs and the significant improvements in 3D human cell culture technologies. The advent of self-organizing 3D structures grown from pluripotent or adult stem cells, so-called organoids, has had a tremendous impact on disease modelling and drug discovery. Embryoids are a multi-lineage type of organoid that does not only mimic a single organ or tissue, but relevant components of the entire human conceptus. Although embryoids are currently still primarily used to investigate early human embryogenesis, their characteristics qualify them as powerful tools for disease modelling and drug discovery in future.

To date, embryoids cannot develop into a human fetus. All existing embryo-like structures discussed above recapitulate particular aspects of peri- and post-implantation development but are far from being equivalent to human embryos. However, since this is a rapidly growing research field and the development of new embryoids, which more closely mimic in vivo human embryos, can be expected, it is important to anticipate putative upcoming ethical concerns. International consensus has already been published demanding a ban of the usage of human embryoids for reproductive purposes, particularly the implantation of embryoids into human uteri. Still, the question of the ethical status of human embryoids must be discussed and appropriate guidelines for the work with synthetic embryo-like structures have to be established [33,97,101,102,119,120].

In future, studies on human embryoids will lead to a more comprehensive understanding of the fundamental biological processes underlying human embryogenesis, including their deregulation in the course of pathologies. For example, gene editing approaches will allow the establishment of innovative embryo models for the identification of relevant genetic factors and for the investigation of the development of specific human genetic diseases. However, all so far existing stem cell-based embryo models harbor different specific limitations and unique properties, mainly due to the fact that they recapitulate distinct developmental stages (Figure 1; Table 1). To date, only the 2D gastrulation model allows the investigation of the development of trophectoderm-like cells [104,105]. Of the three 3D embryoids, only the PASE model can be used to investigate amniotic ectoderm development and the dissemination of primitive streak cells into the surrounding microenvironment. Only PASE represents a 3D asymmetric cyst with a bipolar pattern containing squamous amnion-like cells and columnar pluripotent embryonic disc-like cells, which disseminate upon primitive streak development [107,108,109,110]. In future, 3D gastruloids derived from Chiron-treated hESCs could provide a valuable experimental tool for both, basic research and drug discovery attempts focusing on stages of human embryogenesis that lie beyond the boundaries defined by the legal regulations for ex vivo human embryo cultivation [111].

However, all these different approaches of embryoid development share common shortcomings, when the obtained results are to be translated to the human in vivo condition. The absence of immunological and inflammatory responses that occur in a human body, or the lack of extra-embryonic tissues are such limiting factors, just to name a few. In future, extra-embryonic endoderm stem cells or trophoblast stem cells can possibly be used to further develop the existing models into the direction of the in vivo human condition [9,10,11,92,93,94,95].

A better understanding of early human embryogenesis is of special interest considering the fact that failure of implantation or embryonic development is estimated to occur in up to 50% of pregnancies. Pregnancies often fail in the first two weeks before the woman is aware of the pregnancy. This is the time period in which lumenogenesis and amniogenesis of the epiblast take place [110,121,122,123]. Due to a variety of reasons, including ethical concerns, obvious limitations regarding sample accessibility, the shortcomings of previously available model systems and the well described differences between human and, e.g., rodent embryogenesis, the molecular processes controlling this period of embryo development remained largely elusive. Accordingly, also little is known about the genetic causes or the environmental triggers, such as potentially involved toxins, which could be responsible for failures during human peri-implantation development. In the near future, the use of embryoids will significantly contribute to a more comprehensive picture of the involved underlying developmental processes.

Intriguingly, in future, the here discussed human embryo models could play a pivotal role as “door-openers” for powerful new strategies to identify therapeutic targets and to test new drugs. In particular, human embryo models could allow to draw special attention to the putative teratogenic effects of newly discovered drugs. To evaluate the applicability of embryoids for drug discovery and testing the expression of cell type- or tissue-specific regulators, enzymatic chemical metabolizers and drug transporters must be studied in great detail in these models. Still, one can already foresee that upon future improvements regarding their standardization, reproducibility, and scalability, synthetic human embryoids could provide powerful platforms for high-throughput drug and toxicity screenings.

## Figures and Tables

**Table 1:** Summary of hPSC-based in vitro embryo models.

Embryo Model	Reference	Structure	In Vivo Equivalent	In Vitro Development
Gastrulation micropatterned colony	[104,105]	2D	Gastrulation: germ layer patterning and gastrulation “movements”	hESCs are forced to grow in a confined geometry on micropatterned coverslips; treatment of hESCs with BMP4 triggers self-organized spatial patterns of an outer trophectoderm-like ring, an inner ectodermal circle and a ring of mesendoderm in between, where cells undergo EMT and migrate inwards mimicking gastrulation
Asymmetric early post-implantation epiblast	[106]	3D	Early post-implantation, pre-gastrulation: day 10 human epiblast	hESCs dispersed in hydrogel supplemented with Matrigel form lumenal sacs, which break the anterior–posterior symmetry upon BMP4 treatment and polarize into ectoderm and mesoderm
Post-implantation amniotic sac embryoid (PASE)	[107,108,109,110]	3D	Post-implantation, gastrulation: post-implantation development until days 13–19 (including epiblast cyst formation at days 6–7, asymmetric sac development at days 7–13 and the onset of gastrulation at days 13-19)	hESCs form an amniotic sac, with amniotic ectoderm, an amniotic cavity and an embryonic disc; the embryonic disc develops into a primitive streak-like region with cells undergoing EMT and disseminating into the microenvironment
Gastruloid	[111]	3D	Post-gastrulation: 72 h gastruloids show features of late Carnegie-stage 8 embryos (days 17–19) to early Carnegie stage 9 embryos (days 19–21)	hESCs are treated with Chiron, a WNT agonist, before seeding in low-adherence plates; gene expression studies of the so formed gastruloids reveal evidence for derivatives of the three germ layers organized in a spatiotemporal manner

## Data Availability

Not applicable.
